# Tamock: simulation of habitat-specific benchmark data in metagenomics

**DOI:** 10.1186/s12859-021-04154-z

**Published:** 2021-05-01

**Authors:** Samuel M. Gerner, Alexandra B. Graf, Thomas Rattei

**Affiliations:** 1 Division of Computational System Biology, Department of Microbiology and Ecosystem Science, University of Vienna, Vienna, Austria; 2 Department Bioengineering, University of Applied Sciences FH Campus Wien, Vienna, Austria

**Keywords:** Metagenomics, Simulation, Benchmark, Study design

## Abstract

**Background:**

Simulated metagenomic reads are widely used to benchmark software and workflows for metagenome interpretation. The results of metagenomic benchmarks depend on the assumptions about their underlying ecosystems. Conclusions from benchmark studies are therefore limited to the ecosystems they mimic. Ideally, simulations are therefore based on genomes, which resemble particular metagenomic communities realistically.

**Results:**

We developed Tamock to facilitate the realistic simulation of metagenomic reads according to a metagenomic community, based on real sequence data. Benchmarks samples can be created from all genomes and taxonomic domains present in NCBI RefSeq. Tamock automatically determines taxonomic profiles from shotgun sequence data, selects reference genomes accordingly and uses them to simulate metagenomic reads. We present an example use case for Tamock by assessing assembly and binning method performance for selected microbiomes.

**Conclusions:**

Tamock facilitates automated simulation of habitat-specific benchmark metagenomic data based on real sequence data and is implemented as a user-friendly command-line application, providing extensive additional information along with the simulated benchmark data. Resulting benchmarks enable an assessment of computational methods, workflows, and parameters specifically for a metagenomic habitat or ecosystem of a metagenomic study.

**Availability:**

Source code, documentation and install instructions are freely available at GitHub (https://github.com/gerners/tamock).

**Supplementary Information:**

The online version contains supplementary material available at 10.1186/s12859-021-04154-z.

## Background

To guide researchers in choosing the most appropriate software and workflows for their given study, benchmarking is carried out to compare and evaluate their performance. Typically benchmark studies use simulated data or sequence mock communities with known composition.

However, conclusions of such benchmarks are limited to their underlying data. The benchmark initiative CAMI and Sczyrba et al. [1], Tamames et al. [2] suggested different methods depending on sample properties. To assess method performance and selection for a specific study, benchmark data properties should therefore resemble the actual data of the study as closely as possible. Creating such benchmark data is challenging, since metagenomic communities can vary substantially in complexity and composition including fractions of sequences with unknown origin. Artificial design and resulting biases further limit the scope and power of benchmarks.

The number of metagenomes sequenced with subsequent retrieval of genome draft bins increased drastically in recent years. Pasolli et al. recently uncovered over 150,000 microbial genomes [3]. 77% of species found in this study were never described before, showing the severe lack of reference genomes in current databases despite the massive sequencing efforts undertaken. The underrepresentation of microbial diversity in reference databases will lead to high fractions of unclassified data for metagenomes from understudied environments, however these unclassified and unknown sequence fractions will affect analysis of such metagenomes.

Creating benchmark data limited to known genomes will only provide limited applicability for studies targeting largely unknown habitats, however without a ground-truth available, methods and workflows cannot be fully assessed. To bypass this limitation, we utilize available reference genomes for known fractions of a metagenomic sample while incorporating the unknown sequence fraction into a benchmark sample as well. With this approach we keep original sample complexity while providing a ground-truth in benchmark data sets for method assessment.

We developed Tamock (loosely named from targeted mock communities) to automatically create benchmark data directly based on the taxonomic composition of a metagenomic sample to provide a sample specific benchmark for a particular habitat. We utilize all available information of NCBI RefSeq [4] to simulate all classifiable sequence fractions of a metagenomic sample while keeping the unknown sequence fraction to maintain original sample properties for each benchmark sample. To our knowledge, no other benchmark creation tool provides such habitat-specific benchmarks directly based on real metagenomic samples.

### Comparison to other benchmark data creation tools

Since the advent of genome sequencing, multiple tools to create in silico metagenomic data sets have been published. The aim of metagenomic sequence simulators such as FASTQSim [5], Grinder [6], BEAR [7] or CAMISIM [8] has been primarily the evaluation of novel bioinformatic methods. For such evaluations, complete control over all parameters of a dataset is a prime objective, often resulting in benchmark datasets consisting of considerably fewer genomes compared to environmental metagenomic samples. Metagenomic samples can easily consist of multiple hundred to thousands of species depending on the environment they originate from.

In contrast to previous established tools, Tamock includes the unknown and unclassified sequence fraction for the creation of benchmark data sets. We aim to provide tailored benchmarks (e.g. at the beginning of a bioinformatic study) to evaluate methods and tools for a particular habitat or set of data, hence the aim to mirror original sample complexity in respective benchmark data by the inclusion of unknown sequence fractions. We believe this approach will be most valuable when analysing understudied habitats, for which no comparable benchmark data has been used in respective benchmark efforts. To minimize the barrier for researchers to create benchmark data with limited knowledge about their data at the beginning of a study, Tamock was developed with easy usage in mind, requiring no mandatory user input for parameter optimization or the preparation of reference genome data. Reference genome data is automatically downloaded and prepared the first time a genome is classified in a provided sample.

For the generation of benchmark data, all mentioned tools can use abundance profiles as input to create a metagenomic data set. FASTQSim and BEAR provide the possibility to determine the genome profile directly from a sample. FASTQSim relies on BLAST [9] to search sequences from the original sample against reference genomes which gets computationally very expensive for multiple, large metagenomic datasets. BEAR applies RAPSearch [10] for homology-based inference of abundance profiles using protein sequences from RefSeq, limiting the profile generation only to protein-coding sequences. To improve on these limitations, Tamock applies Centrifuge, which can classify all sequences from a metagenomic sample requiring substantially less computational resources to generate an abundance profile based on a complete sample. Both Grinder and CAMISIM do not include the creation of abundance profiles directly from a metagenomic sample but focus on extended downstream options such as generation of transcriptomic, proteomic or 16S rRNA sets for Grinder or in silico replicates, time series or differential abundance samples based on various distributions for relative abundances for CAMISIM.

The approach of Tamock has been utilized to assess assembly and binning methods for the analysis of urban metagenomes, providing researchers guidelines for the analysis of urban metagenomic data [11]. We present the use of benchmark samples created by Tamock for the evaluation of assembly and binning methods as an example use case for selected urban metagenomes and samples from the Integrative Human Microbiome Project [12] (Additional file 2: Table S1).

## Implementation

Tamock uses taxonomic abundances obtained by classifying sequences from a metagenomic sample to define the taxonomic profile for the creation of benchmark data.

### Taxonomic profile

Tamock determines the taxonomy of all sequences by applying Centrifuge [13], a resource-efficient, *k-mer* based taxonomic profiler. The use of Centrifuge enables Tamock to run on a standard desktop machine due to its low memory requirements. By default, an index for prokaryotic, human, and viral sequences (p + h + v) provided by the Centrifuge authors is used. Additional indexes as well as custom indexes can be created or are available from Centrifuge.

### Selection of reference genomes

Sequence counts classified at species or strain level are used to create the profile for a benchmark sample. All sequence reads classified to taxonomic species level or below (i.e. strain level) are assigned to a reference genome from NCBI RefSeq while reads classified to higher taxonomic levels (genus and higher) are not simulated as they cannot be assigned to a single reference genome.

Sequences with multiple taxonomic assignments are counted proportionally (counts for a taxon increased by one third for a sequence classified to three different taxa). Depending on the reference database, sequences assigned to a strain might not have a corresponding reference genome in NCBI RefSeq. To incorporate such sequences, they are reassigned as follows:

1. All sequences assigned to sub-species level without a corresponding reference genome found in NCBI RefSeq are added to their respective species counts.
2. All species counts are assigned to strains of the same species with a corresponding reference genome already present.

**Fig. 1:**
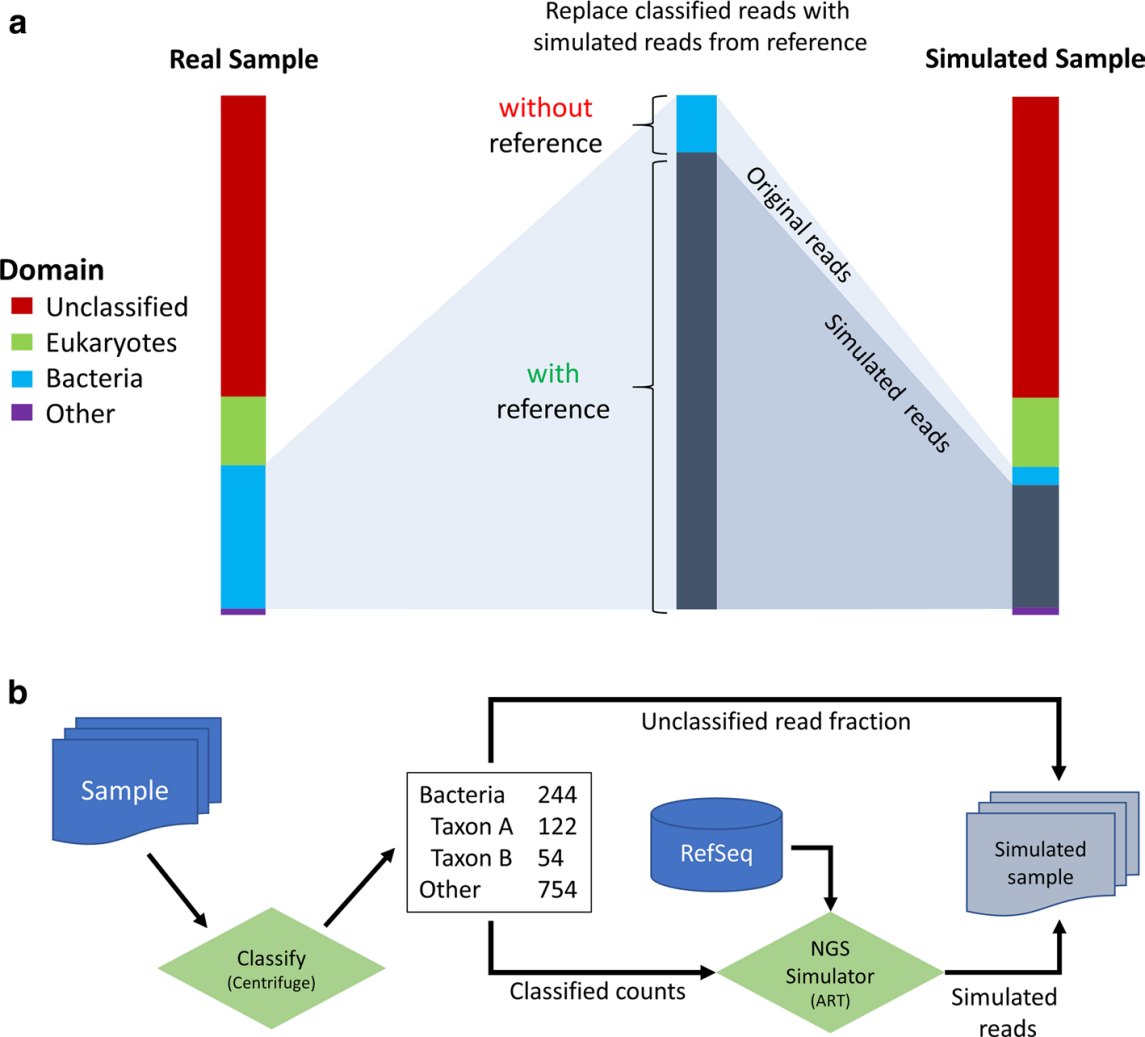
Tamock design and workflow. **a** Creation of benchmark data by Tamock simulating the bacterial sequence fraction. By default, Tamock only simulates the bacterial fraction, however Tamock is also able to include or simulate only Eukaryota, Viruses or Archaea to the extent of available reference genomes. **b** Workflow of Tamock to create tailored in silico benchmark data. Classified reads are replaced by simulated reads of equivalent abundance from reference genomes, while unclassified or reads without a reference are kept to maintain original sample complexity

Sequence counts from species to strain level are distributed using the ratio of already assigned sequence counts of all strains of the respective species. Ultimately, all sequences from strains without reference genomes or classified to species level are assigned to a specific reference genome. This strategy accounts for the taxonomic profile of the sample while optimizing for all genomes present in the reference database, closely maintaining the original complexity (Fig. 1a).

### Benchmark data creation

Benchmark sequences are simulated from the taxonomic profile with according abundances. Tamock replaces all sequences classified and matched to reference genomes from the original sample with simulated sequences (see Additional file 2: Table S1 for metrics). By default, only the bacterial domain is simulated, however other domains such as Eukaryota, Archaea or Viruses can be simulated as well as far as reference genomes are present in NCBI RefSeq.

Sequencing simulation is performed by ART [14]. The resulting simulated sequence collection is combined with all unclassified sequences for the final benchmark sample (Fig. 1b). Thereby, the benchmark sample reflects the original sample while providing exact sequence counts for the classified sequence fraction as a ground truth for further analysis. Through this process, effects of e.g. read depth, error rates, species and subspecies diversity can be explored for real metagenomic communities. Additional, report files with information for all abundances, classification results and selected reference genomes are provided together with the final benchmark data.

Tamock learns and applies parameters such as sequence error profile, read length, and sequencing depth directly from the input sample by default since the aim is to reproduce the original sample as close as possible. Nonetheless, these parameters can be changed if the user wants to create benchmark data with differing characteristics to conduct experiments to improve experimental design of a study.

## Results and discussion

To highlight the benefit of applying Tamock for study design and method selection, we showcase the use of benchmark samples created by Tamock (v1.3.0). For genome-centric analysis of metagenomes, assembly and binning methods are frequently applied to extract metagenomic bins, resulting in ever-increasing numbers of novel genomes reconstructed from a metagenomic sample. We use Tamock benchmark data to assess the quality of results from an assembly and binning experiment, analysing contamination and presence of tRNA and rRNA genes as described in the MIMAG standards for high (HQ), medium (MQ) and low quality (LQ) metagenome-assembled genome bins [15], while following the workflow from Pasolli et al. [3].

**Fig. 2:**
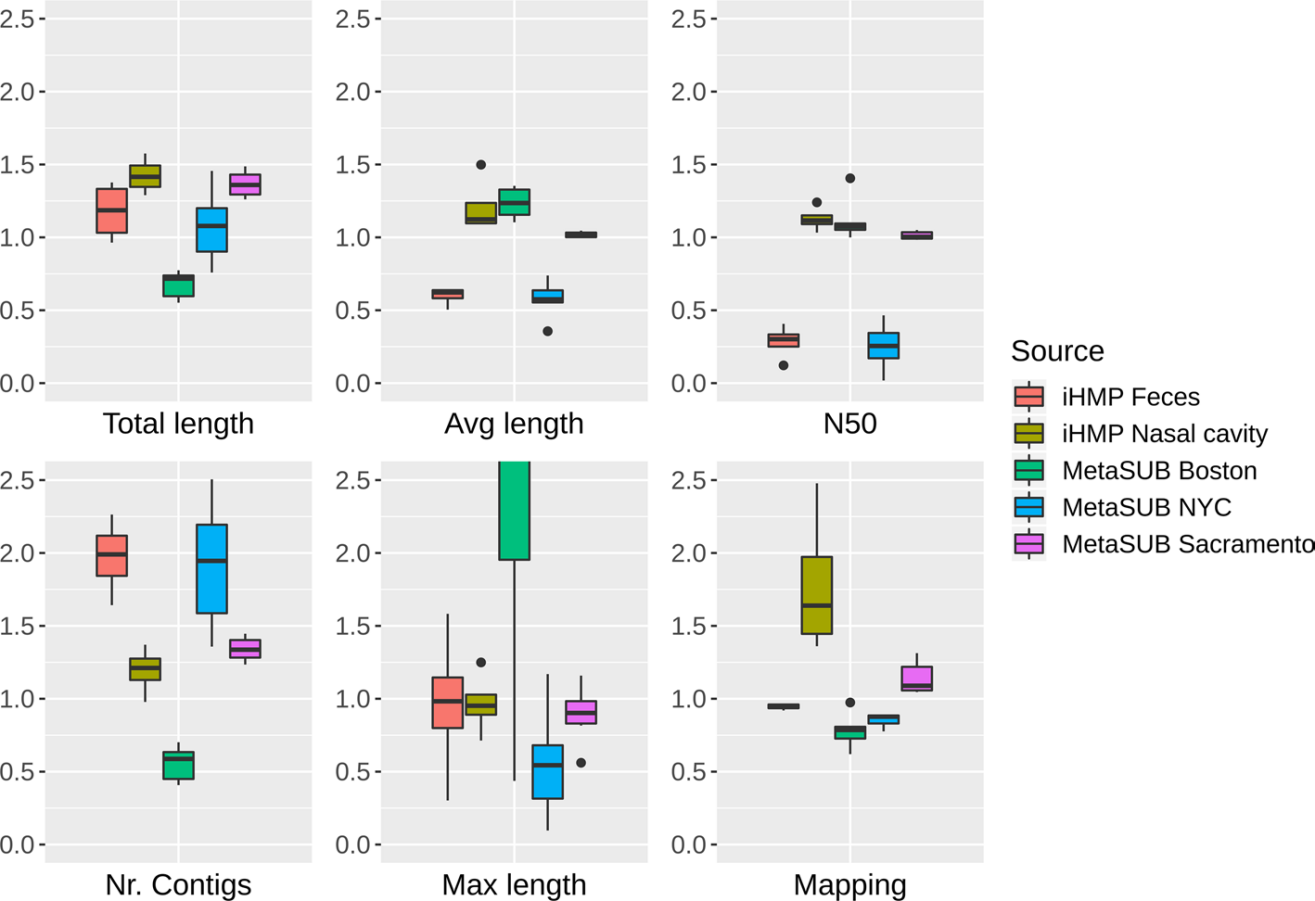
Relative change of assembly performance. The relative change of assembly statistics from original samples to their corresponding simulated samples is shown. A value of 1 displays no change, whereas a value below 1 represents lower values in benchmark samples as well as a value above 1 represents higher values for respective assembly statistics compared to the corresponding original samples. Fold changes are shown for the total, average, and maximum length as well as number of contigs together with N50 value and percentage of reads mapping back to the assembly. Figures were produced using the packages ggplot2 v3.3.0 [25], reshape2 v1.4.4 [26], gridExtra v2.3 [27] in R v3.6.3 [28]

We created benchmark samples for 18 urban metagenome samples from the MetaSUB Consortium [16–18] as well as 8 human microbiome samples from the integrative Human Microbiome Project (iHMP) [12]. All original samples and their corresponding simulated samples by Tamock were assembled using MetaSPAdes v3.13.1 [19] with default parameters. Relative changes in assembly performance from original to their corresponding Tamock benchmark samples (“simulated” samples) are shown in Fig. 2.

Assembled sequences were subsequently binned by MetaBAT v2.15 [20]. Following the MIMAG standards [15], we used CheckM v1.1.2 [21] for completeness and contamination values, barrnap [22] to predict rRNA genes and tRNA-Scan-SE v1.3.1 [23] for tRNA genes (Additional file 1: Methods).

High-quality (HQ) genome bins are required to fulfil the following requirements: > 90% completeness and < 5% contamination as well as the presence of 5S, 16S and 23S rRNA genes together with at least 18 different rRNA genes. Medium-quality bins (MQ) are only required to fulfil ≥ 50% completeness and < 10% contamination whereas all remaining bins are classified as low quality (LQ) following MIMAG standards.

**Fig. 3:**
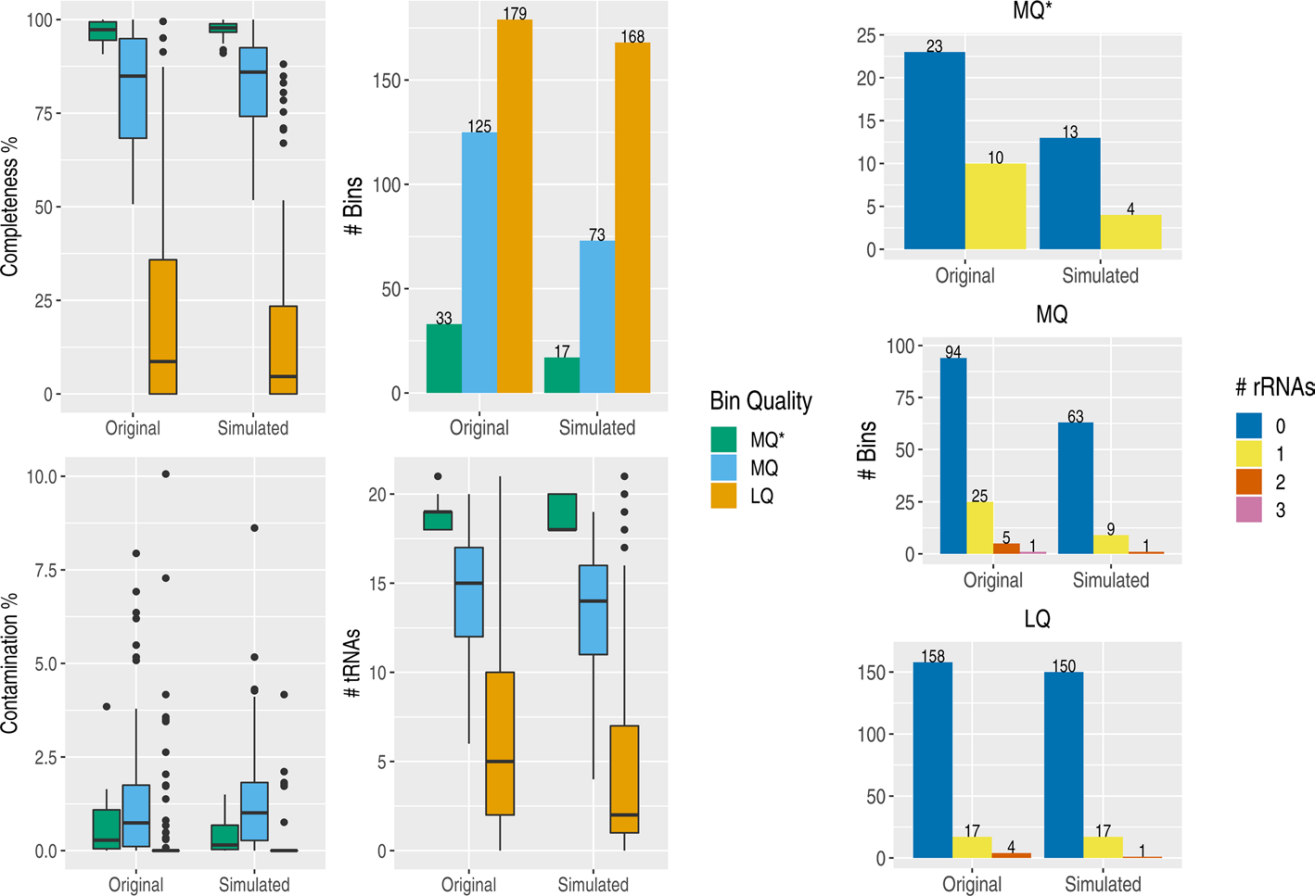
Binning statistics for all genome bins from original and simulated samples. Contamination and completeness values, number of bins as well as tRNA and rRNA genes for all low quality (LQ), medium quality (MQ) and near-high quality (MQ*) bins from both original and Tamock benchmark samples (“Simulated”) are shown. The lack of rRNA genes is prominent in low as well as medium and near-high quality genome bins. Figures were produced using the packages ggplot2 v3.3.0 [25], reshape2 v1.4.4 [26], gridExtra v2.3 [27] in R v3.6.3 [28]

No genome bins with high-quality (HQ) could be assembled and binned from both original as well as corresponding simulated samples. Resulting genome bins did not reach HQ due to the lack of all three rRNA genes present in the genome bins as required by MIMAG standards. One single bin did contain a copy of a 5S, 16S and 23S rRNA gene respectively, however due to insufficient completeness this genome bin only met the requirements for MQ. Genome bins fulfilling all criteria for HQ except the presence of rRNA genes were labelled as MQ* (Fig. 3).

The number of genome bins dropped from a total of 337 to 258 from original to simulated samples.

Due to incomplete reference sequence databases, especially for strains present in a sample but not in reference databases, alterations from an original sample towards its corresponding simulated sample are to be expected and unavoidable. Depending on the composition of a sample, mainly two effects can be observed.

(I) If a sample contains multiple different strains for which only one reference genome is available, classified sequences will be replaced only from a single genome. This is most prominent for samples from MetaSUB Boston. All samples from this group represent urban metagenomes with high fractions of sequences classified to *Homo sapiens* (27 to 81%, Additional file 2: Table S1). As urban metagenomes harbour human sequences from multiple individuals, classification to and resampling from a single reference genome reduces assembly complexity and leads to a reduced total length and number of contigs due to reduced strain variance as well as a higher maximum length of contigs (Fig. 2).
(II) However, if a strain is present in a sample with no direct, but a closely related reference genome in a database, only a subset of sequences will be classified to the respective reference genome with subsequent sampling, resulting in two strains (original and reference) present in the simulated where only one strain has been present in the original sample. Faecal samples from iHMP as well as MetaSUB samples from New York City (NYC) have consistently high fractions of sequences classified as bacterial (34 to 92%) as well as in average 89% sequences assigned to a reference genome (Additional file 2: Table S1). Multiple strains will not be present in a database but will be classified to closely related reference genomes and therefore increase strain diversity and assembly complexity in the simulated sample, leading to reduced average length of contigs and N50 values as well as an increase in the number of contigs (Fig. 2).

Ultimately, the aim of Tamock is to create benchmark data as similar to the original sample as possible by mirroring sample composition, sequence errors, depth and length while replacing the classified sequence fraction with sampled sequences from reference genomes to provide a ground truth for experiments. Despite the inherent limitations as described above for sampling genomes from known reference genomes for unknown metagenomes, most assembly parameters were stable over various metagenomic habitats, reflected in all variables near the value 1 for the comparison of assembly statistics from original and corresponding simulated samples in Fig. 2.

Results from simulated samples with available ground-truth for all sampled reference genomes can subsequently be utilized to assess methods and workflows of interest for an individual study. The abundance, genome coverage, and source for all resampled genomes in a simulated sample provide the base to assess a study design under realistic conditions based on the actual data of interest.

Applied to the presented show case, we can determine the genome coverage required to extract genome bins without any misassemblies compared to their reference genome. We applied MetaQUAST v5.0.2 [24] to assess any potential misassemblies in extracted genome bins. Tables with all sampled genomes and the corresponding abundance are available in the output of Tamock. We were able to successfully extract genome bins without any misassemblies, such as from the iHMP2 stool sample J00827 for *Bifidobacterium adolescentis* ATCC.

The extracted genome bin in the simulated and original data of sample J00827 (Additional file 3: Table S2) only failed HQ standards due to the lack of rRNA genes. In the corresponding simulated sample for J00827, sequences from *B. adolescentis* ATCC were sampled at 9 × coverage for the reference genome and the extracted genome bin covered 89.73% of the reference genome with 1.39% of all reads mapping to the respective genome bin (Additional file 4: Figs. S1–S4).

### Comparison to benchmark data without unknown sequence fraction

The creation of benchmark data for metagenomic samples from complex environments can lead to high numbers of reference genomes required. Multiple hundred to thousands of reference genomes quickly lead to extensive computational resources required, either by long runtimes or high memory usage. The most recent developed CAMISIM, which has been used to create benchmark data for the CAMI Challenge [1], requires several hundred GB of RAM for higher numbers of genomes according to the documentation of CAMISIM.

In contrast to CAMISIM, relative abundances in Tamock are drawn directly from the unknown sample by classifying all sequences with Centrifuge. The usage of Centrifuge and subsequent processing also enables Tamock to process multiple thousands of reference genomes on a standard desktop due to the low memory usage of Centrifuge for indexes i.e. of RefSeq, with runtimes of a few hours for a sample with about 20 Mio sequences and about 4.000 reference genomes excluding the download time of reference genomes (only required once).

To enable a comparison with benchmark data created by current tools such as CAMISIM without inclusion of the unknown sequence fraction, we created benchmark data only from the classified sequence fraction with reference genomes available. Three versions of benchmark data were created. One set of benchmark data consisted only of sequences which are simulated by Tamock and used to replace all corresponding sequences in the original sample (“simonly” benchmark, Additional file 2: Table S1). The subset of original sequences which Tamock replaces with “simonly” is used to create the benchmark data set “orig-repl” for direct comparison with “simonly”. For the third set of benchmark data, the number of sequences which are simulated by Tamock are scaled to the sequence depth of the original sample, i.e. for a sample with 1500 reads of which 1000 reads are classified and assigned to a genome while the remaining 500 reads are unclassified, the second data set multiplied all counts by 1.5 × to create a sample with 1500 reads using relative abundances from the taxonomic profile of classified sequences (“simscaled” benchmark, Additional file 2: Table S1). The functionality to scale the number of classified sequences to a set sequence depth while maintaining relative abundances is available in Tamock via the option “-rn-sim”. This alters sequence depth and composition of the simulated sample compared to the original sample but enables users to create benchmark data for further experiments with different characteristics (see Additional file 1: Methods).

CAMISIM, representing the most current benchmark creation tool, allows taxonomic profiles to be used for benchmark data creation and utilizes ART as well to simulate Illumina sequences. A benchmark data set from Tamock consisting only of the classified sequence fraction with known abundances and a reference genome therefore is the equivalent of a benchmark dataset created by CAMISIM or other tools creating benchmark data from a taxonomic profile with set abundances which is provided by Tamock. No tool to our knowledge deduces the abundance profile directly from a metagenomic sample, simulates and replaces the known sequence fraction while maintaining the characteristics of the original sample by learning parameters such as sequence errors, length and depth for simulation from the original sample and keeping the unknown sequence fraction.

We assembled and binned the “orig-repl”, “simonly” and “simscaled” benchmark in the same manner as described above for original and simulated samples by Tamock. Since the unknown sequence fraction is not part of the benchmark, we observe a loss of total assembly length and number of contigs for all samples with substantial fractions of unclassified data as expected. This is most prominent for samples from MetaSUB Sacramento and iHMP Feces with only 35.4 and 43.4% of all sequences classified on average (Additional file 4: Fig. S5) and can be explained with the lower sequence depth using only the known sequence fraction. However, even in “simscaled” samples with the same sequence depth but reduced sample complexity, we observe a drop in total sequence length for MetaSUB Boston and NYC whereas Sacramento shows a strong increase (Additional file 4: Fig. S6). Comparing only the two benchmark data sets “orig-repl” and “simonly”, the sequence fractions which Tamock effectively exchanges to create a simulated Tamock benchmark sample, assembly statistics such as maximum length, total length and number of contigs increased from “orig-repl” to “simonly”. Since classification of sequences is incomplete, the loss of the unknown sequence fraction will lead to the loss of fractions of sequences for assembly, whereas sampling from a reference genomes for the exact same sequence depth as done for all counts in “orig-repl” to create “simonly” will improve assembly performance. As described above, this will lead to a slight reduction in sample complexity, particularly for assembly. This is especially true for samples with large fractions of eukaryotic sequences such as from iHMP Nasal Cavity, where e.g. for sequences classified to human only one reference genome is sampled (Additional file 4: Fig. S7).

Samples with fairly high fractions of classified data such as from MetaSUB NYC with 78.2% of sequences classified in average show a drop in total assembly length for purely simulated benchmark data, whereas the simulated sample by Tamock showed only a slight increase in total length compared to the assembly of original samples (Fig. 2). The loss of total assembly length indicates that substantial parts of the unknown sequence fraction did belong to partially classified sequences, leading to the observed loss of sequence coverage during assembly, particularly for MetaSUB NYC samples.

**Fig. 4:**
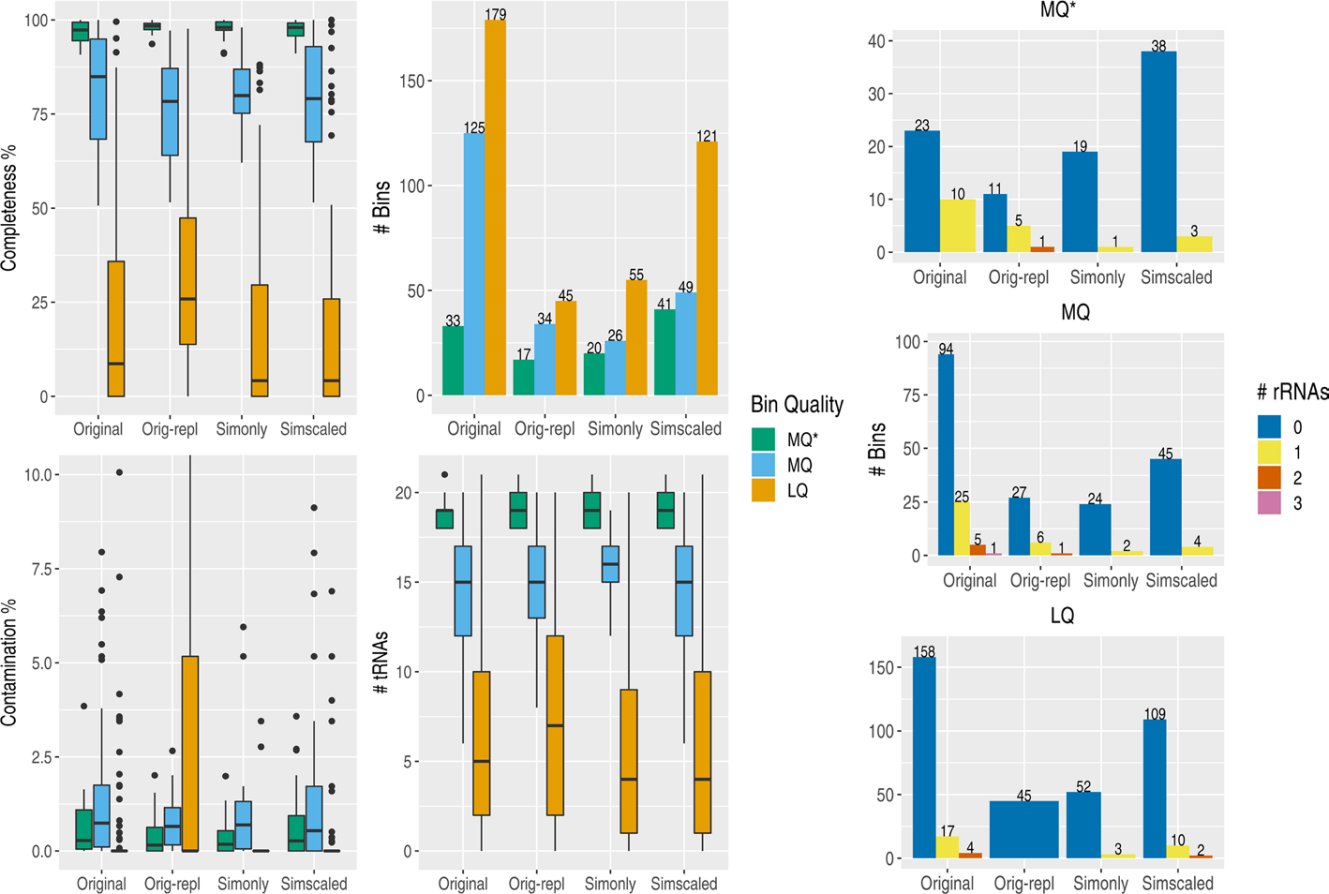
Binning statistics for original and all simulated data sets without unknown sequence fractions. Contamination and completeness values, number of bins as well as tRNA and rRNA genes for all low quality (LQ), medium quality (MQ) and near-high quality (MQ*) bins from original samples, original samples with only the classified sequence fraction “orig-repl”, “simonly” as well as “simscaled” benchmarks with only simulated sequences are shown. Despite less sample diversity, the lack of rRNA genes is even more prominent in both purely simulated benchmark data sets. Figures were produced using the packages ggplot2 v3.3.0 [25], reshape2 v1.4.4 [26], gridExtra v2.3 [27] in R v3.6.3 [28]

Since a substantial part of the original sample is missing, there is a considerable loss in the number of resulting genome bins in purely simulated benchmarks. The complete “simonly” dataset is sampled from reference genomes, as such sample diversity is lowered, reducing overall assembly and binning difficulty, shown by the less severe loss of near high-quality genome-drafts (MQ*), however the total number of resulting genome bins dropped strongly from 337 from the original samples to 101 for “simonly” (Fig. 4). This is very close to the number of resulting genome bins from “orig-repl” with 96 bins, supporting the exchangeability of these two sequence fractions as performed by Tamock to create benchmark data. A slight increase of both MQ* and LQ quality genome bins is to be expected due to the both the likely loss of non-classified sequences belonging to a genome with classified sequences while reducing assembly complexity due to the selection of reference genomes for replacement. Assembly complexity can be reduced by e.g. the merging of closely related strains into one reference genome if only one is present in the reference database.

For “simscaled” samples, a slight increase in genome draft bins of MQ* quality can be observed, which is based on the increased coverage by scaling up all counts. Nonetheless, the diversity of the original samples is still lost and even with less complexity, there is a drop in the total number of resulting genome bins from 337 to 211.

Ultimately, benchmark samples created by Tamock showed performance closest to the original samples in both assembly and binning performance, underlying the importance to include unknown sequence fractions for metagenomic data with unclassified data. Considering only the respective sequence fractions Tamock alters in an original sample creating a simulated sample by Tamock, we could show the consistent number of resulting genome bins for only these sequence fractions, supporting the approach of Tamock to replace all classified sequences. Benchmark data based on only the known sequence fraction from a metagenome sample provided results deviating further from corresponding results of original samples as Tamock benchmark data.

## Conclusion

Benchmark data created by Tamock can be used to quickly evaluate a workflow for a novel metagenomic dataset, compare and evaluate methods, as well as to improve the interpretation for any results from metagenomic studies, since assumptions for the quality of results can be easily tested and evaluated. Particularly for studies analysing novel or extreme habitats, we believe Tamock to be of high value for informed study design and formulation of a hypothesis with realistic expectations for the quality of results prior to an experiment.

To our knowledge, Tamock is the only benchmark data creation tool which enables researchers to simulate samples directly from an original sequence file without any further input or action in form of required parameter settings, reference data preparation or other time-consuming preparation steps. Tamock creates habitat-specific benchmark data for metagenomic samples. Resulting benchmark data sets can be used to assess any metagenomic workflow or method for a particular study, providing researchers with performance assessments on their individual research question and data.

## Availability and requirements

- Project name: Tamock.
- Project home page: https://github.com/gerners/tamock.
- Operating systems: Linux and MacOS.
- Programming language: Perl.
- Other requirements: Perl >  = v5.12.0; GNU Scientific Library (GSL).
- License: GNU GPL 3.0.
- Any restriction to use by non-academics: None.

## Supplementary Information

**Additional file 1: Supplementary Figure 1**. Genome fractions for the top five Bifidobacterium strains classified in the iHMP2 stool sample J00827 (Benchmark data set) for which contigs of the genome bin 36 could by aligned are shown. The reference genome GCF_000010425.1 corresponds to Bifidobacterium adolescentis ATCC 15703 with 89.73% coverage and has been sampled 9x in the simulated sequence fraction constituting for 1.39% of all reads. The figure is produced with MetaQUAST v5.0.2 (Mikheenko et al. 2016).

**Additional file 2: Supplementary Figure 2**. Percentage of sequence reads mapping to the respective reference genome for the top five Bifidobacterium strains classified in the iHMP2 stool sample J00827 (Benchmark data set). 1.36% of sequences mapped to the reference genome GCF_000010425.1 (Bifidobacterium adolescentis ATCC 15703) while 1.39% of sequences mapped back to genome bin 36. The figure is produced with MetaQUAST v5.0.2 (Mikheenko et al. 2016).

**Additional file 3: Supplementary Figure 3**. The number of misassemblies for genome bin 36 compared to the top five Bifidobacterium strains classified in theiHMP2 stool sample J00827 (Benchmark data set) is shown. No misassemblies were identified for all five reference genomes. The figure is produced with MetaQUAST v5.0.2 (Mikheenko et al. 2016).

**Additional file 4: Supplementary Figure 4**. The total length of aligned contigs for genome bin 36 compared to the top five Bifidobacterium strains classified in theiHMP2 stool sample J00827 (Benchmark data set) are shown. The total aligned length of all contigs sums up to 1875 kbps, constituting for 89.73% genome coverage for GCF_000010425.1 (Bifidobacterium adolescentis ATCC 15703). The figure is produced with MetaQUAST v5.0.2 (Mikheenko et al. 2016).

## Data Availability

The source code is available on GitHub (https://github.com/gerners/tamock), sources for the presented example data are available in Additional file 2: Table S1.
