# Supplementary material for: Tamock: simulation of habitat-specific benchmark data in metagenomics
Source: BMC Bioinformatics. 2021 May 1;22:227. doi: 10.1186/s12859-021-04154-z (PMC8088724; doi:10.1186/s12859-021-04154-z)
Supplement: Supplementary file 4 — Additional file 4: Supplementary Figure 4. The total length of aligned contigs for genome bin 36 compared to the top five Bifidobacterium strains classified in theiHMP2 stool sample J00827 (Benchmark data set) are shown. The total aligned length of all contigs sums up to 1875 kbps, constituting for 89.73% genome coverage for GCF_000010425.1 (Bifidobacterium adolescentis ATCC 15703). The figure is produced with MetaQUAST v5.0.2 (Mikheenko et al. 2016). [file 12859_2021_4154_MOESM4_ESM.pdf]

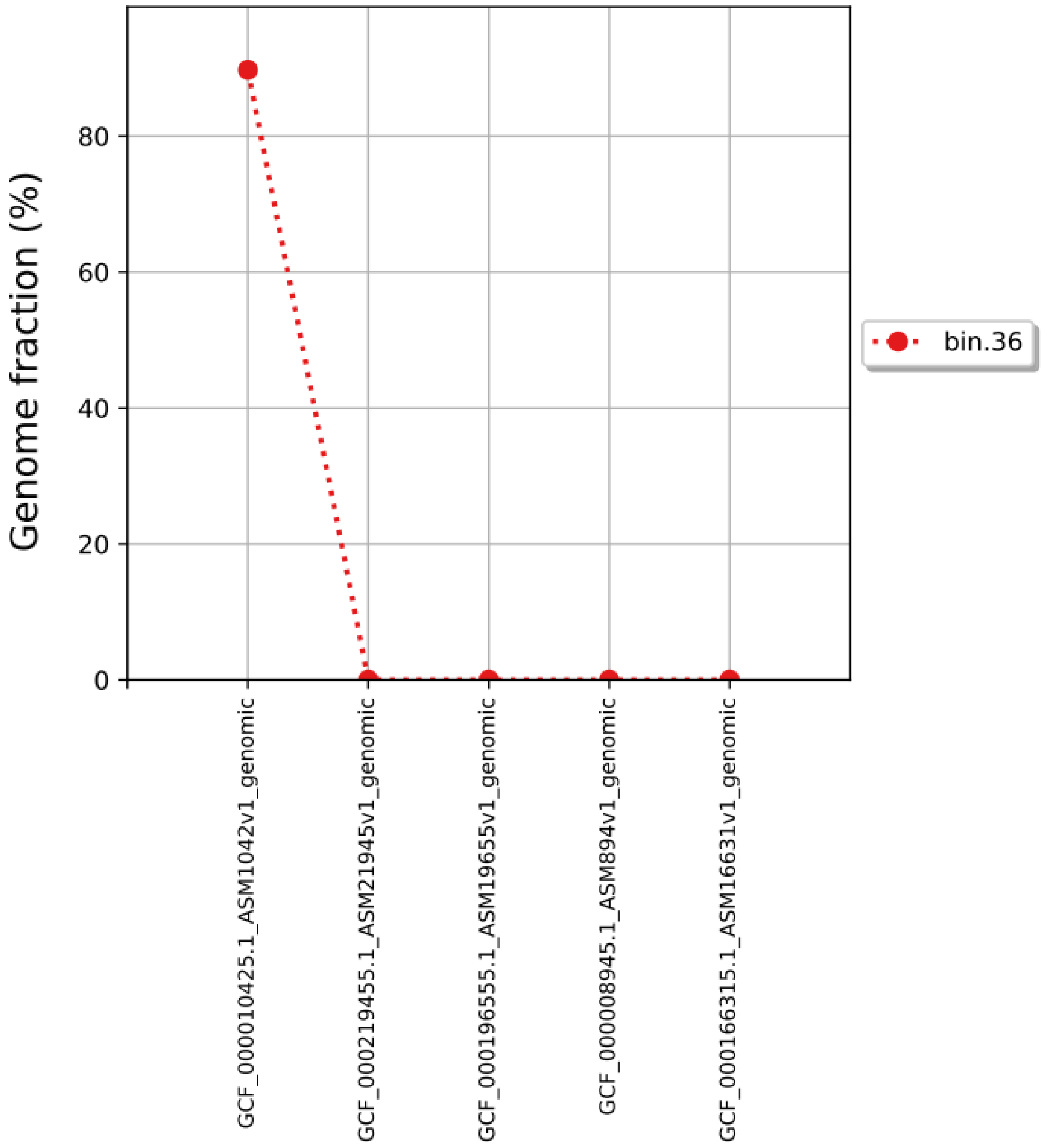

Supplementary Figure 1

Genome fractions for the top five *Bifidobacterium* strains classified in the iHMP2 stool sample J00827 (Benchmark data set) for which contigs of the genome bin 36 could by aligned are shown. The reference genome GCF_000010425.1 corresponds to *Bifidobacterium adolescentis ATCC 15703* with 89.73% coverage and has been sampled 9x in the simulated sequence fraction constituting for 1.39% of all reads.

The figure is produced with MetaQUAST v5.0.2 (Mikheenko et al., 2016).

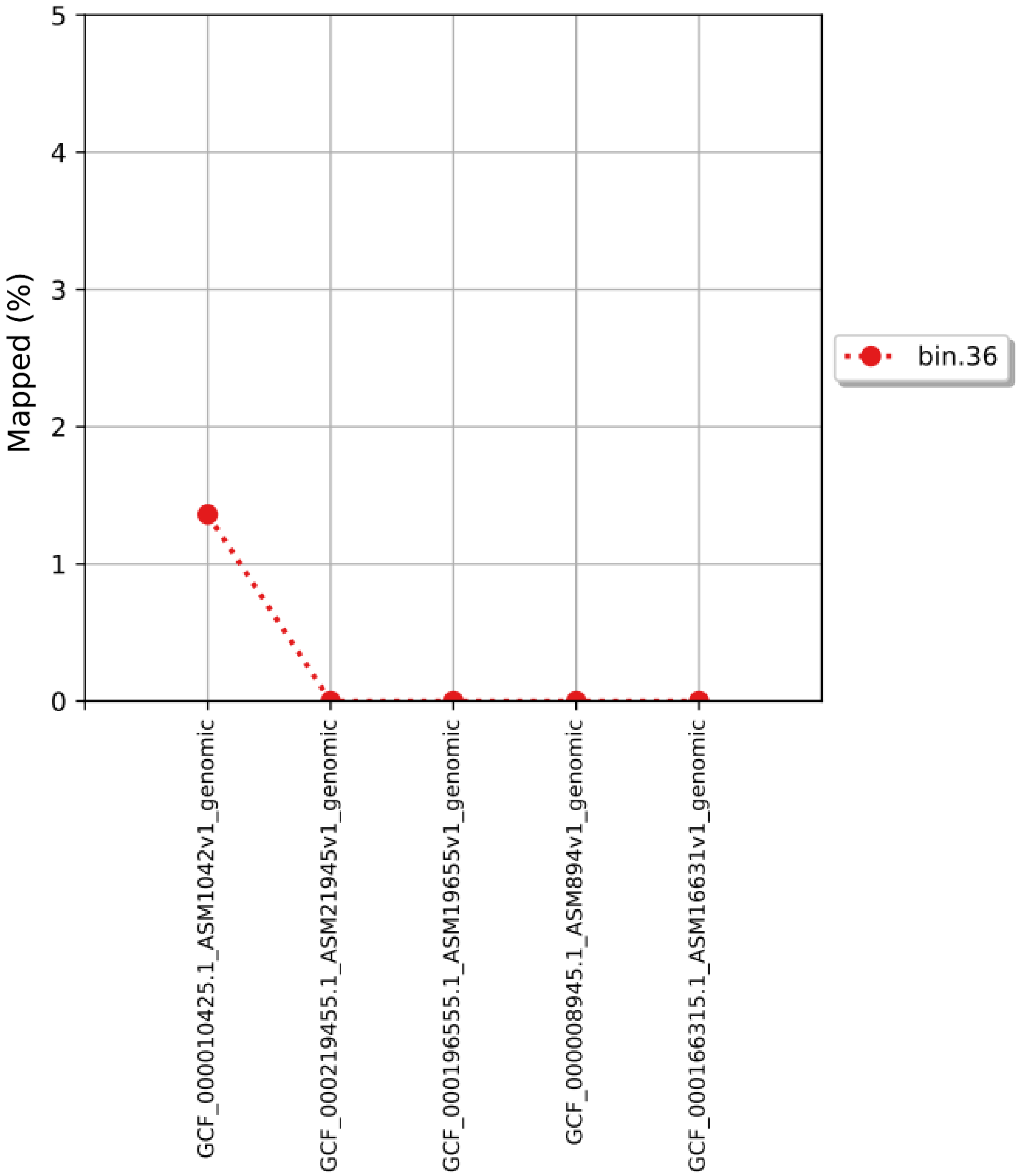

Supplementary Figure 2

Percentage of sequence reads mapping to the respective reference genome for the top five *Bifidobacterium* strains classified in the iHMP2 stool sample J00827 (Benchmark data set). 1.36% of sequences mapped to the reference genome GCF_000010425.1 (*Bifidobacterium adolescentis ATCC 15703)* while 1.39% of sequences mapped back to genome bin 36.

The figure is produced with MetaQUAST v5.0.2 (Mikheenko et al., 2016).

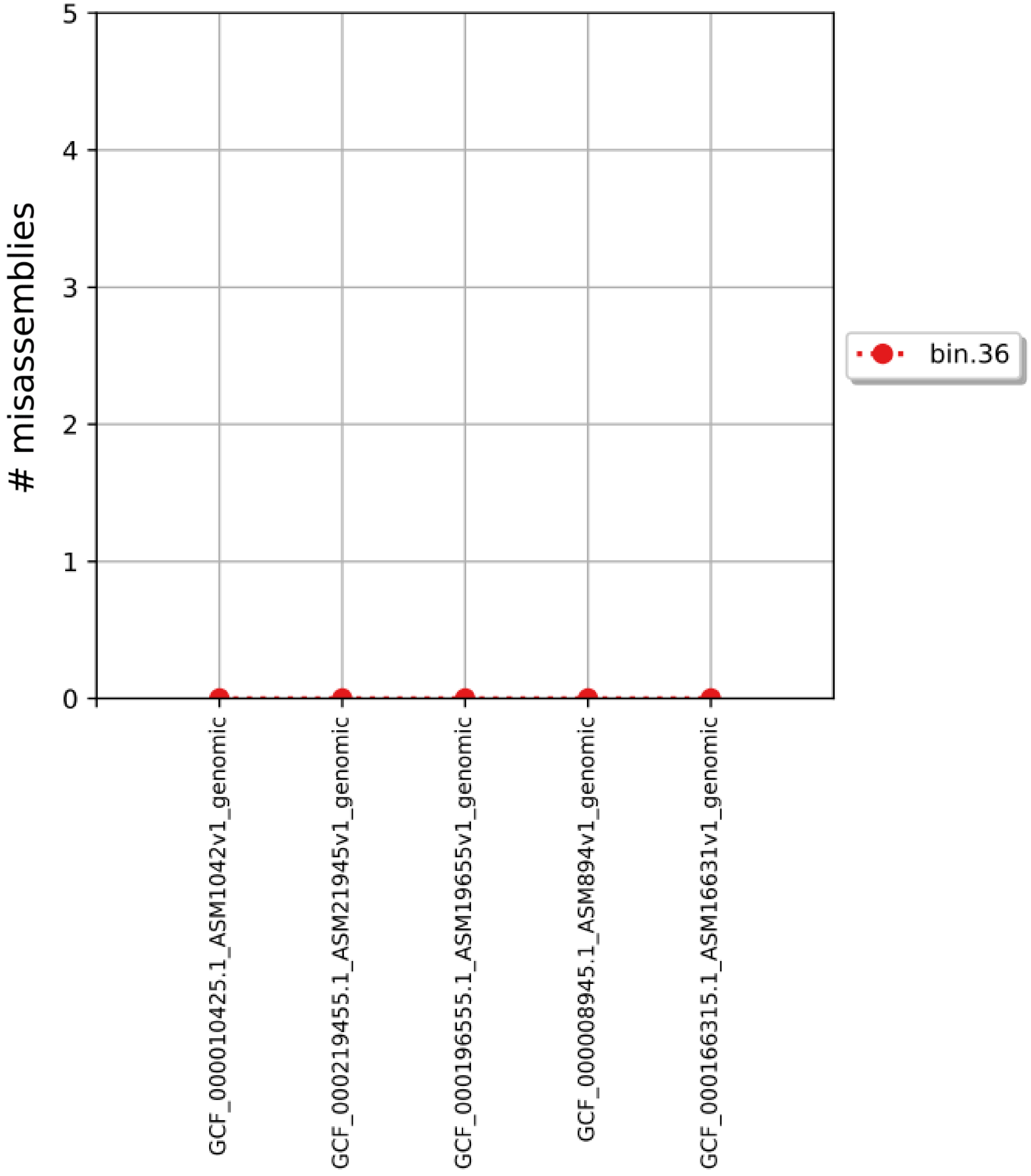

Supplementary Figure 3

The number of misassemblies for genome bin 36 compared to the top five *Bifidobacterium* strains classified in the iHMP2 stool sample J00827 (Benchmark data set) is shown. No misassemblies were identified for all five reference genomes.

The figure is produced with MetaQUAST v5.0.2 (Mikheenko et al., 2016).

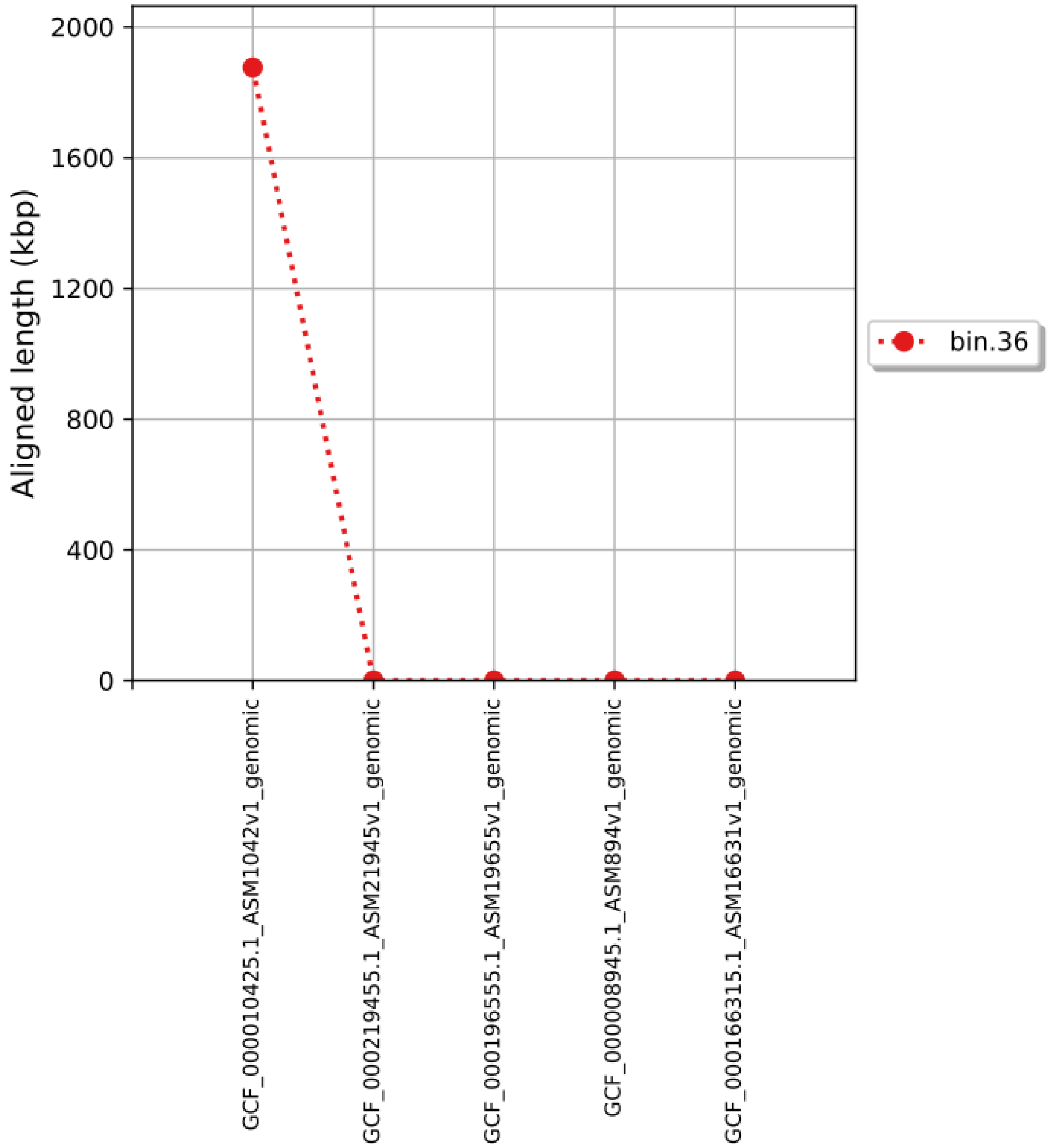

Supplementary Figure 4

The total length of aligned contigs for genome bin 36 compared to the top five *Bifidobacterium* strains classified in the iHMP2 stool sample J00827 (Benchmark data set) are shown. The total aligned length of all contigs sums up to 1875 kbps, constituting for 89.73% genome coverage for GCF_000010425.1 (*Bifidobacterium adolescentis ATCC 15703).*

The figure is produced with MetaQUAST v5.0.2 (Mikheenko et al., 2016).

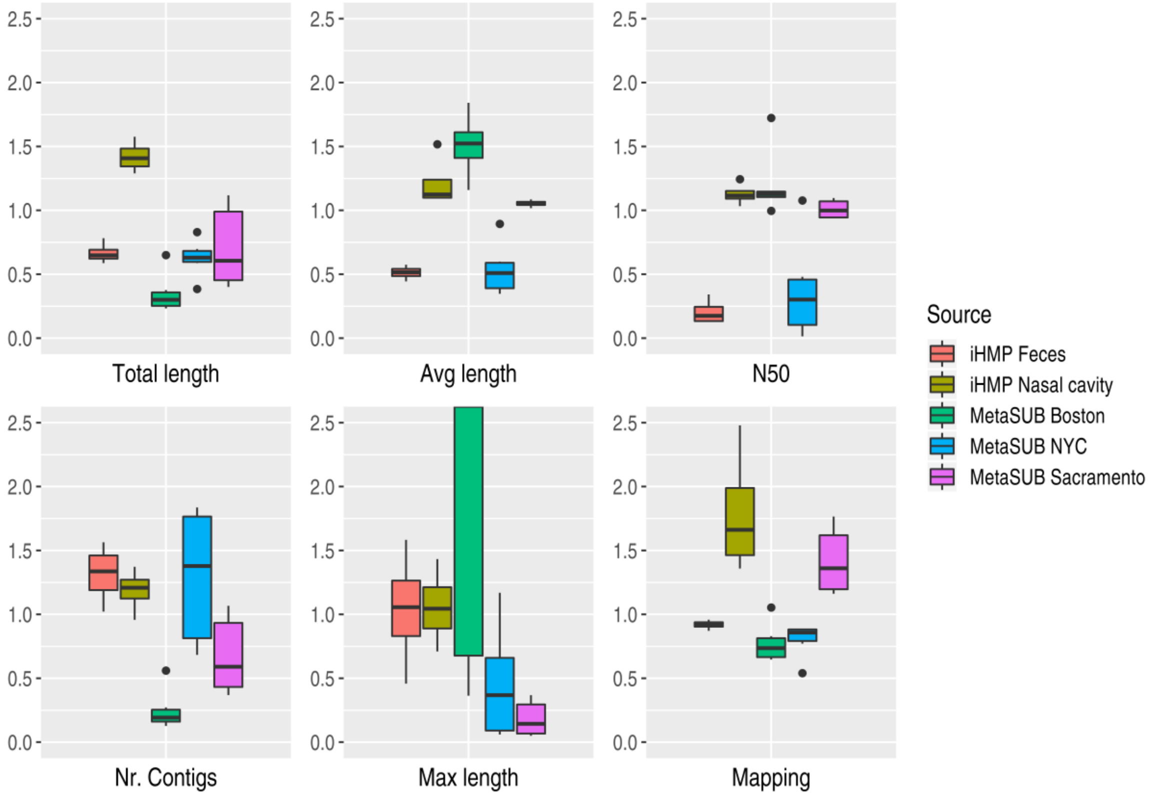

Supplementary Figure 5 – Original vs "simonly" relative change of assembly performance

The relative change of assembly statistics from original samples to their corresponding benchmark samples with only the simulated sequence fraction ("simonly") is shown. A value of 1 displays no change, whereas a value below 1 represents lower values in benchmark samples as well as a value above 1 represents higher values for respective assembly statistics compared to the corresponding original samples. Fold changes are shown for the total, average and maximum length as well as number of contigs together with N50 value and percentage of reads mapping back to the assembly.

Total length in "simonly" samples drops strongly compared to full Tamock Benchmarks (Figure 2) for all samples with substantial fractions of unclassified data (MetaSUB Sacramento and iHMP Feces with only 35.4 and 43.4% of all sequences classified on average). The same is true for the number of contigs after assembly and particularly maximum length for MetaSUB NYC and Boston samples.

Figures were produced using the packages ggplot2 v3.3.0 (Wickham, 2016), reshape2 v1.4.4 (Wickham, 2007), gridExtra v2.3 (Auguie, 2017) in R v3.6.3 (R Core Team, 2020).

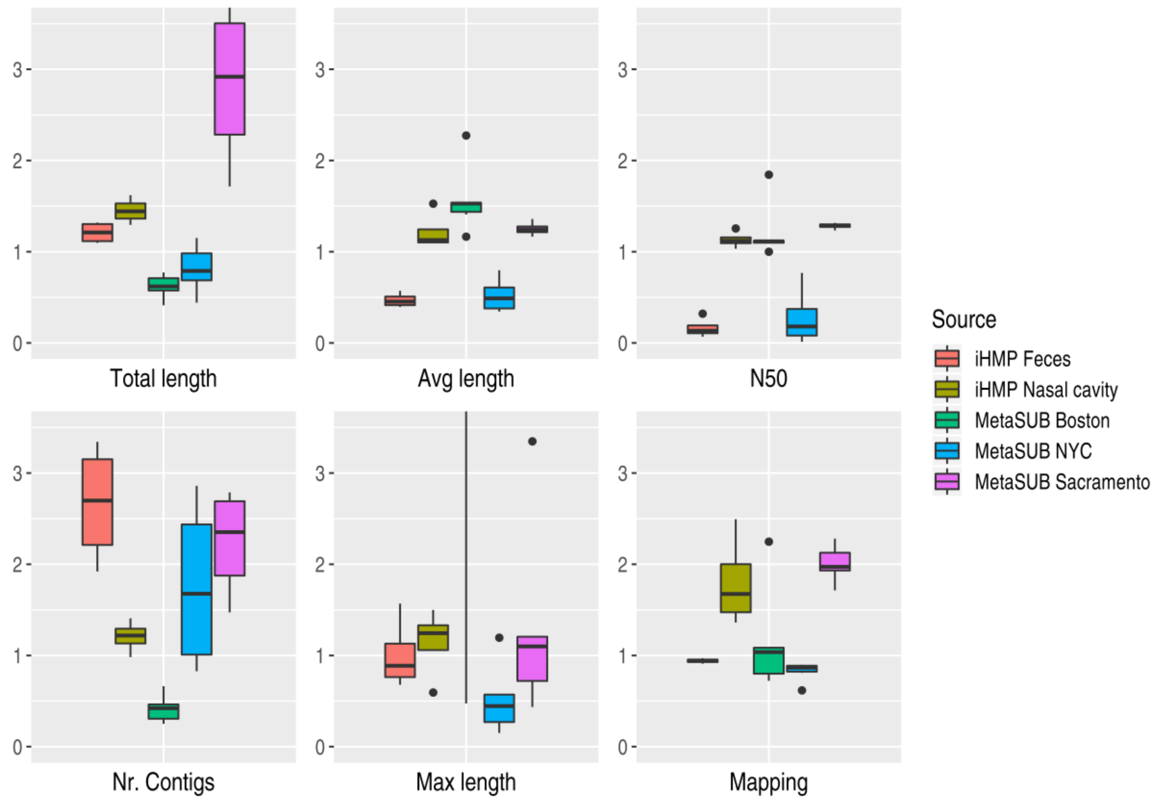

Supplementary Figure 6 – Original vs “simscaled” relative change of assembly performance

The relative change of assembly statistics from original samples to their corresponding benchmark samples with only the simulated sequence fraction but with scaled up sequence counts to the total number of all sequences in original samples (“simscaled”) is shown. A value of 1 displays no change, whereas a value below 1 represents lower values in benchmark samples as well as a value above 1 represents higher values for respective assembly statistics compared to the corresponding original samples. Fold changes are shown for the total, average and maximum length as well as number of contigs together with N50 value and percentage of reads mapping back to the assembly.

Even though sample complexity is reduced due to the absence of the unknown sequence fraction, average and maximum contig length is still reduced in e.g. MetaSUB NYC. Overall, assembly statistics deviate further from the performance of original samples than Tamock benchmark data (Figure 2).

Figures were produced using the packages ggplot2 v3.3.0 (Wickham, 2016), reshape2 v1.4.4 (Wickham, 2007), gridExtra v2.3 (Auguie, 2017) in R v3.6.3 (R Core Team, 2020).

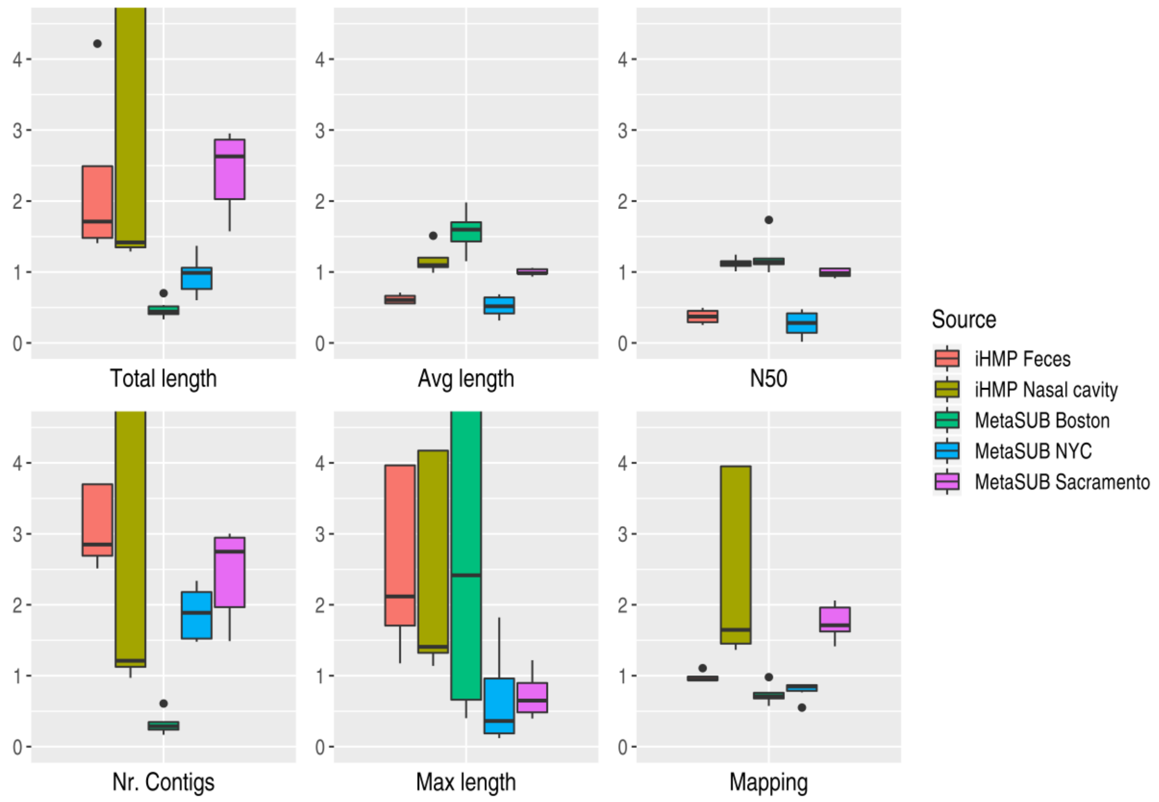

Supplementary Figure 7 – "orig-repl" vs "simonly" relative change of assembly performance

The relative change of assembly statistics from the subset of original sequences replaced by simulated sequences ("orig-repl") and the corresponding simulated sequences ("simonly") is shown. A value of 1 displays no change, whereas a value below 1 represents lower values in benchmark samples as well as a value above 1 represents higher values for respective assembly statistics compared to the corresponding original samples. Fold changes are shown for the total, average and maximum length as well as number of contigs together with N50 value and percentage of reads mapping back to the assembly.

Assembly parameters such as total length, number of contigs and maximum length are increased in the "simonly" assemblies. This is expected, as particularly for iHMP Nasal activity, the consolidation of all human sequences into one reference genome selected for "simonly" reduced sample complexity for assembly. For samples with large bacterial fractions (e.g. MetaSUB Boston, NYC), sample complexity will be reduced as well due to the lack of reference genomes for every strain, leading to presumably less complexity compared to the original sample, where some sequences might be assigned to closely related species and therefore reduce the number of genomes represented in "simonly" for assembly.

Figures were produced using the packages ggplot2 v3.3.0 (Wickham, 2016), reshape2 v1.4.4 (Wickham, 2007), gridExtra v2.3 (Auguie, 2017) in R v3.6.3 (R Core Team, 2020).
